# Use of professional practice guidance resources in pharmacy: a cross-sectional nationwide survey of pharmacists, intern pharmacists, and pharmacy students

**DOI:** 10.1186/s40545-021-00395-8

**Published:** 2021-12-29

**Authors:** Deanna Mill, Jacinta L. Johnson, Kenneth Lee, Sandra M. Salter, Danielle D’Lima, Liza Seubert, Rhonda Clifford, Amy T. Page

**Affiliations:** 1 School of Allied Health, The University of Western Australia, Perth, WA Australia; 2 UniSA Clinical and Health Sciences, University of South Australia, Adelaide, SA Australia; 3 SA Pharmacy, SA Health, Adelaide, SA Australia; 4 Centre for Behaviour Change, Department of Clinical, Educational and Health Psychology, University College London, London, UK; 5 Pharmacy Department, The Alfred, Melbourne, VIC Australia; 6 Centre for Medicines Use and Safety, Monash University, Melbourne, VIC Australia

**Keywords:** Pharmacist, Practice standards, Code of ethics, Practice guidelines, Professional behaviour

## Abstract

**Background:**

Variations in practice are commonplace in healthcare where health professionals, such as pharmacists act as autonomous practitioners. This is evident in simulated patient studies, where pharmacists practice does not meet widely accepted standards for medicines supply or treatment of an ailment. To promote best pharmacy practice a myriad of guidance resources including practice guidelines, codes and standards are produced by professional organisations. These resources provide a framework for pharmacy practice and endeavour to facilitate consistency in provision of pharmacy-based services to consumers. Despite their role in specifying essential pharmacist behaviours, there is limited research exploring if and how these resources are used in practice.

**Objective:**

To characterise Australian pharmacists’ use of the Pharmaceutical Society of Australia’s Code of Ethics, Professional Practice Guidelines and Professional Practice Standards.

**Methods:**

A cross-sectional, self-administered, electronic survey of registered pharmacists, intern pharmacists and pharmacy students living in Australia was conducted in July 2020. Questions considered use of professional practice resources (by resource group) in the preceding 12 months. Data were analysed descriptively.

**Results:**

Of 601 responses included in the analysis 462 (76.9%) of respondents were registered pharmacists, 88 (14.6%) pharmacy students and 51 (8.5%) intern pharmacists. Interns and students accessed overarching practice resources, such as the Professional Practice Standards, Code of Ethics and Dispensing Practice Guidelines more frequently than practising pharmacists. Pharmacists accessed professional practice guidelines, such as Practice Guidelines for the Provision of Immunisation Services Within Pharmacy, more often than students. More pharmacists than interns and students indicated that they would access guidelines to resolve practice and patient care issues. All resources except the Professional Practice Standards for Pharmacists (67.4%) were accessed by less than 50% of respondents in the preceding 12-month period. Reasons for not accessing resources varied between participant and resource groups, and generally were due to a lack of awareness of the resource or not considering them necessary for the individual’s practice.

**Conclusion(s):**

Access and use patterns for professional practice guidance resources change with experience. Professional organisations responsible for developing resources should consider these patterns when designing and reviewing resources and related policies. To ensure resources are meeting the needs of the profession, students, interns, and pharmacists should be involved in the review of and design of further resources.

## Abstract (article highlights)

- Pharmacy students access core guidance resources more than practising pharmacists.
- Pharmacists and interns access service specific resources more than students.
- Most resources were accessed by less than half of respondents in a 12-month period.
- Resources used to update knowledge, check practice and improve resource familiarity.
- Irrelevant content, no perceived need for information and poor awareness limited use.

## Background

Practice standards, codes of conduct and practice guidelines exist to communicate the expected minimum conduct of pharmacists when they are providing care to patients [1–4]. These key professional guidance resources allow professional and regulatory bodies to communicate the legal, ethical and professional requirements that must be adhered to when a pharmacist provides patient care [1–3]. Professional practice guidance resources differ regarding their specific aims and purpose. 'Codes’ typically dictate high level principles that should be considered by the professional regardless of area of practice (e.g., do no harm) [2, 4], ‘practice standards’ communicate expected behaviours of the profession (e.g., provide medicines information to all patients when dispensing their prescriptions) [1] and ‘practice guidelines’ provide specific advice around the steps needed to provide a medicine or service (e.g., vaccination service) [3, 5].

Given pharmacists are autonomous professionals some variations in provision of care are expected. Previously published simulated patient scenario studies, descriptions of disciplinary hearings and studies exploring pharmacists' navigation of ethical scenarios highlight that these inconsistencies, when compared to practice recommended in guidance resources, can lead to suboptimal patient care [6–16]. Such suboptimal practice and professional transgressions have included inappropriate storage and supply of scheduled medicines (e.g., without adequate medicines counselling), inappropriate disposal of medicines and inappropriate referrals to other health professionals [6–16]. Adhering to the recommendations in these practice guidance resources should enable pharmacists to ensure they provide, consistent, quality services to all patients. Practice guidelines function to educate pharmacists to enable consistent service provision, and also to ensure that service provision to patients is evidenced based, safe, effective, and adheres to legal and ethical practice requirements [3, 17]. These resources can provide guidance to pharmacists when they do not know how to proceed in a specific situation or need to clarify their responsibilities. Professional practice guidance resources can also be used to help individuals reflect on their own practice, to provide guidance in the provision of services, for the public to understand what they can expect from a pharmacist, to outline the expectations of providing a service that is reimbursed by the government or another funder, and occasionally in instances of investigating malpractice [1, 4]. Professional practice guidance resources are, therefore, generally introduced to pharmacy students and intern pharmacists as part of their education and professional socialisation.

Given professional practice guidance resources are essential for ensuring quality pharmaceutical care, the International Pharmaceutical Federation have recommended their member associations make them a priority to ensure pharmacists in their respective countries have access to adequate guidance [18, 19]. The Pharmaceutical Society of Australia (PSA), the peak national body representing Australian pharmacists, continually develop professional practice guidance resources for the pharmacy profession. These resources are usually developed in collaboration with or endorsed by government agencies, regulatory authorities and other relevant organisations (e.g., the Pharmacy Guild of Australia or Consumers Health Forum of Australia) [1, 5, 20]. These organisations invest considerable time and money to develop these professional practice guidance resources, with the intention that they will support consistency and quality in practice and service provision by Australian pharmacists.

While professional practice resources are intended to be key in guiding pharmacy practice, this relies on them being used. Furthermore, use of practice guidance resources is a professional behaviour in its own right, yet little is known about if and how professional practice resources are used by pharmacists. One previous qualitative study on the application of ethical principles by Australian pharmacists (*n* = 25) found that most participants did not look to the Code of Ethics for Pharmacists when faced with an ethical dilemma, but instead relied on their own knowledge and the principle of acting in ‘the best interests of the patient’ [7]. It was concluded that pharmacists found the Code of Ethics document was ‘of little value in practice’ [7]. Another qualitative study conducted over a decade ago found Australian pharmacists (*n* = 17) displayed a poor awareness and limited use of the PSA Practice Standards for Dispensing, citing its length and lack of identification of essential versus desirable actions as problematic [16]. The authors discovered a lack of integration of practice standards into practice processes and as such called for the useability and applicability of these documents to be reviewed to optimise integration to improve processes and patient care [16]. Since this study the Professional Practice Standards for Pharmacists have been reviewed and updated twice, however, internationally no further research exploring pharmacists’ use of the standards, or any other related professional practice guidance resources has been identified [16].

Beyond the work of Hattingh et al. [16], no previous studies have investigated if and how pharmacists, interns or pharmacy students use professional practice resources more broadly. Without contemporary clarity as to the current usage patterns of professional practice resources, the ability for policymakers and developers to improve and tailor them to the needs of the profession is limited. Furthermore, understanding usage patterns may partly explain observed and reported inconsistencies in practice. Thus, the present study aimed to characterise Australian pharmacists’ use of general professional practice guidance resources including nationally recognised Practice Guidelines [3, 5, 17, 21–30], Code of Ethics [2] and Professional Practice Standards for Pharmacists [1].

## Methods

### Study design

A cross sectional, self-administered, electronic survey of pharmacists, intern pharmacists and pharmacy students living in Australia was conducted in July 2020. The survey considered professional practice resources essential to guiding general professional, ethical and legal practice when delivering pharmacy services and practising as a pharmacist. Only guidance resources that are freely accessible by pharmacists, intern pharmacists, and pharmacy students (including practice guidelines [3, 5, 17, 21–30], Code of Ethics [2] and Professional Practice Standards for Pharmacists [1]) were examined (Box 1). They are particularly relevant to pharmacists practising in a community setting, although not all guidance resources are directly relevant to every pharmacist’s current scope of practice (e.g., they may not currently administer vaccinations; therefore, vaccination guidelines are not necessary).

**Box 1:** Pharmaceutical Society of Australia (PSA) Professional Practice Guidance Resources included in survey by resource group

Professional practice guidance resource grouping for survey	Professional practice guidance resources included
Resource Group 1—Overarching Practice Standards, Codes and Guidelines (Referred to intext as ‘Overarching Resources’)	My Health Record Guidelines for Pharmacists [29] Clinical Governance Principles for Pharmacy Services [27] Dispensing Practice Guidelines [3] Code of Ethics for Pharmacists [2] Professional Practice Standards for Pharmacists [1] Guide to Providing Pharmacy Services to Aboriginal and Torres Strait Islander People [23]
Resource Group 2—Community Pharmacy Core Professional Services Practice Guidelines (Referred to intext as ‘Core Professional Service Resources’)	Guidelines for Pharmacists Providing Dose Administration Aid Services [26] Guidelines for Pharmacists Providing Staged Supply Services [24] Guidelines for Pharmacists Providing Medscheck and Diabetes Medscheck Services [25] Practice Guidelines for the Provision of Immunisation Services Within Pharmacy [5] Guidelines for the Continued Dispensing of Eligible Prescribed Medicines by Pharmacists [28]
Resource Group 3—Accredited Medication Review Services Practice Guidelines (Referred to intext as ‘Medication Review Resources’)	Guidelines for Quality Use of Medicines (QUM) Services [30] Guidelines for Pharmacists Providing Home Medicines Review (HMR) Services [21] Guidelines for Pharmacists Providing Residential Medication Management Review and QUM Services [22] Guidelines for Comprehensive Medication Management Reviews [17]

This survey was conducted as part of a broader research project seeking to understand Australian pharmacists’ information seeking behaviours and use of a wide range of professional practice guidance resources including: practice standards, codes of conduct, practice guidelines, medicines supply guidelines and the Australian Pharmaceutical Formulary and Handbook [31]. The professional practice guidance resources included in the full study were chosen in collaboration with the peak body representing Australian pharmacists, the Pharmaceutical Society of Australia (PSA). Findings relating to information seeking behaviour, use of medicines supply guidelines and the Australian Pharmaceutical Formulary and Handbook will be reported elsewhere.

The conduct and results of this study are reported according to the Checklist for Reporting Results of Internet E-Surveys (CHERRIES) checklist (Table 4 in Appendix) [32].

### Ethical approval

Approval to conduct this study was granted by The University of Western Australia (UWA) Human Research Ethics Committee in June 2020 (RA/4/20/6014).

### Participants

Pharmacists and intern pharmacists were eligible to participate if they held current registration with the Australian Health Practitioner Regulation Agency in Australia. Pharmacy students were eligible to participate if they were enrolled in a pharmacy degree course accredited by the Australian Pharmacy Council and currently studying in Australia.

### Sample size

A minimum 1% quota sample of pharmacists, intern pharmacists and pharmacy students were prespecified and calculated using Pharmacy Board registration data that was current at the time [33]. The selection of 1% of the population was intended to be pragmatic and achievable in the absence of existing research to inform the selection of relevant outcomes to inform a sample size calculation. Furthermore, there was no reliable method for researchers to contact all pharmacists registered in Australia to recruit a systematic or randomised sample. At the time of designing this study, there were 32,777 registered Australian pharmacists, thus a minimum of 328 registered pharmacists were recruited. There were 1865 provisionally registered pharmacists in Australia; therefore, a minimum of 19 intern pharmacists were recruited. There were approximately 6500 pharmacy students in Australia; therefore, a minimum of 65 pharmacy students were recruited.

### Recruitment/distribution

The survey was open from 7th July 2020 to 31st July 2020. All initial contact with participants was via the internet. A description of the study and Qualtrics™ (Qualtrics, Provo, UT, Version July 2020 to September 2020) survey link and/or QR code was shared via professional organisations email lists (Pharmaceutical Society of Australia and Society of Hospital Pharmacists Australia), on social media (pharmacy related Facebook groups, Twitter, LinkedIn, pharmacy related Instagram pages) via advertisements in pharmacy related print and electronic media (Pharmacy Daily, Australian Journal of Pharmacy), through sharing of emails with intern training program providers, directors of pharmacy programs, pharmacy student associations, pharmacy banner groups, individual pharmacies and through the research team’s professional networks. The advertisements encouraged participation and sharing of the opportunity to participate. The link directed participants to the full participant information landing page and consent to participate page prior to allowing them to complete the screening questions.

### Incentive to participate

Upon completion of the survey participants could elect to enter a draw to win one of three $100 retail vouchers. Details provided by the participants were collected using a separate Qualtrics survey link and could not be linked to the participant’s responses.

### Survey design

The survey was developed by the research team and reviewed by the Pharmaceutical Society of Australia (PSA) Projects team. Draft questions were piloted via the platform (Qualtrics™) for readability, content and platform useability by a convenience sample of pharmacists (*n* = 12), intern pharmacists (*n* = 4) and pharmacy students (*n* = 6) invited via the research teams’ professional network. In response to pilot feedback minor changes were made to response options, display of the survey, and typographical errors were corrected.

### Final survey

The full survey comprised a total 83 items including multiple-answer checkbox, ranking, multiple-choice, and free text response questions that considered the participants information seeking behaviour and use of professional practice guidance resources in the past 12 months. Questions pertaining to the current study (use of professional practice resources) broadly examined resource selection, frequency of use, reasons resources were selected (or not selected for use), how resources were used, perceived usefulness of resources, and key demographics. Adaptive questioning was enabled based on the participant’s individual responses. Response items were randomised to reduce bias in responses. The professional practice resources examined in the current study were separated into three groups, namely, ‘Overarching Resources’, ‘Core Professional Service Resources’ and ‘Medication Review Resources’ (Box 1).

Mandatory response was enabled for all questions except the free text responses and multiple answer checkbox questions, where no selection indicated that none of the choices were appropriate. An ‘I don’t know’ or ‘I can’t remember’ response was available for questions, where it was deemed appropriate. The survey program prompted participants to complete mandatory questions prior to submitting that page. Participants could choose to go back through questions and change responses prior to submitting the final survey. The survey was an open survey available nationally using online survey software system, Qualtrics™. All responses were anonymous.

Participation in the survey was voluntary and consent to participate was indicated through response to a question at the beginning of the survey. All participants were made aware of the purpose of the study, time needed to complete the survey, data storage, investigators and funder through the participant information available at the beginning of the survey. Thus, providing informed consent.

The ‘prevent ballot box stuffing’ function was turned on in the Qualtrics™ program and enabled placement of a cookie on the access page for the survey link, preventing participants taking the survey multiple times. Qualtrics™ anonymise responses was enabled, so IP addresses were not recorded or checked. No log file analysis techniques were used. Qualtrics™ bot detection was enabled.

### Data analysis

The data set was exported from Qualtrics™ to the latest version of Microsoft Excel. Responses that had the demographics section of the survey and the first content question answered/completed were eligible for inclusion for this analysis. This included partial responses. Responses that met this criterion but were detected with an atypical timestamp for completion (e.g., less than 3 min, when it is estimated to take at least 5 min to get to the first content question) were reviewed to determine if the survey had been completed and presented as intended in the software.

Descriptive statistics including counts and proportions were calculated in STATA Software, Release 16 (StataCorp LLC, College Station, TX, USA). Unique site visitors could not be tracked in Qualtrics™; therefore, view rate and participation rate are not reported. No statistical correction was utilised in this analysis.

## Results

After screening, 601 (*n* = 601/774, 77.6%) responses met the minimum requirement for inclusion in the current analysis and are presented in this report. Of these, 462 (*n* = 462/601, 76.9%) respondents were registered pharmacists, 88 (*n* = 88/601, 14.6%) were pharmacy students and 51 (*n* = 51/601, 8.5%) were intern pharmacists. Table 1 summarises the demographics of the survey respondents.

**Table 1:** Demographics of survey respondents

Characteristic	Registered pharmacist (*N* = 462)	Pharmacy intern (*N* = 51)	Pharmacy student (*N* = 88)	All (*N* = 601)
Gender (*n* (%))				
Male	132 (28.6)	12 (23.5)	24 (27.3)	168 (28.0)
Female	328 (5.3)	39 (76.5)	63 (71.6)	430 (71.5)
Non-conforming/gender variant	0 (0.0)	0 (0.0)	0 (0.0)	0 (0.0)
Prefer not to answer	2 (0.4)	0 (0.0)	1 (1.1)	3 (0.5)
Age (in years) (*n* (%))				
18–24	18 (3.9)	39 (76.5)	70 (79.5)	127 (21.1)
25–34	215 (46.5)	10 (19.6)	12 (13.6)	237 (39.4)
35–44	101 (21.9)	1 (2.0)	4 (4.5)	106 (17.6)
45–54	61 (13.2)	1 (2.0)	1 (1.1)	63 (10.5)
55–64	47 (10.2)	0 (0.0)	0 (0.0)	47 (7.8)
65 +	18 (3.9)	0 (0.0)	0 (0.0)	18 (3.0)
Prefer not to answer	2 (0.4)	0 (0.0)	1 (1.1)	3 (0.5)
None of the above	0 (0.0)	0 (0.0)	0 (0.0)	0 (0.0)
State of workplace/study (*n* (%))				
New South Wales	85 (18.4)	6 (11.8)	13 (14.8)	104 (17.3)
Victoria	118 (25.5)	9 (17.6)	19 (21.6)	146 (24.3)
Queensland	54 (11.7)	11 (21.6)	19 (21.6)	84 (14.0)
South Australia	46 (10.0)	10 (19.6)	12 (13.6)	68 (11.3)
Western Australia	127 (27.5)	7 (13.7)	18 (20.5)	152 (25.3)
Northern Territory	5 (1.1)	2 (3.9)	0 (0.0)	7 (1.2)
Australian Capital Territory	10 (2.2)	3 (5.9)	3 (3.4)	16 (2.7)
Tasmania	12 (2.6)	3 (5.9)	3 (3.4)	18 (3.0)
Prefer not to answer	4 (0.9)	0 (0.0)	1 (1.1)	5 (0.8)
Currently member of any pharmacy organisations (*n* (%))*				
Pharmaceutical Society of Australia	233 (50.4)	34 (66.7)	80 (90.9)	347 (57.7)
Society of Hospital Pharmacists Australia	126 (27.3)	15 (29.4)	54 (61.4)	195 (32.4)
Pharmacy Guild of Australia	93 (20.1)	9 (17.6)	26 (29.5)	128 (21.3)
Professional Pharmacists Australia	49 (10.6)	6 (11.7)	4 (4.5)	59 (9.8)
National Australian Pharmacy Student Association (NAPSA)	11 (2.4)	9 (17.6)	43 (48.9)	63 (10.5)
International Pharmaceutical Federation (FIP)	12 (2.6)	3 (5.9)	2 (2.3)	17 (2.8)
None of the above^#^	88 (19.0)	7 (13.7)	4 (4.5)	99 (16.5)
Prefer not to answer^#^	5 (1.1)	0 (0.0)	0 (0.0)	5 (0.8)
Other	43 (9.3)	1 (2.0)	2 (2.3)	46 (7.7)
Pharmacist principal role (*n* (%))				
Community pharmacy, owner	48 (10.4)	–	–	–
Community pharmacy, employee	209 (45.2)	–	–	–
Hospital pharmacy	110 (23.8)	–	–	–
Academia	25 (5.4)	–	–	–
Consultant	33 (7.14)	–	–	–
Industry	8 (1.7)	–	–	–
Prefer not to answer	3 (0.7)	–	–	–
Other	26 (5.6)	–	–	–
Intern pharmacist principal place of practice (*n* (%))				
Community Pharmacy	–	30 (58.8)	–	–
Hospital Pharmacy	–	19 (37.3)	–	–
Industry	–	0 (0.0)	–	–
Prefer not to answer	–	0 (0.0)	–	–
Other	–	2 (3.9)	–	–
Pharmacist years registered (*n* (%))			–	–
0–2	56 (12.1)	–	–	–
3–5	77 (16.7)	–	–	–
6–10	114 (24.7)	–	–	–
11–20	108 (23.4)	–	–	–
21–30	41 (8.9)	–	–	–
> 31	66 (14.3)	–	–	–
Current medication management review accreditation (*n* (%))				
Yes	95 (20.6)	–	–	–
No	366 (79.2)	–	–	–
Prefer not to answer	1 (0.2)	–	–	–
How often have they worked as a sole pharmacist in the last 12 months (*n* (%))				
Never	90 (19.5)	–	–	–
Rarely	115 (24.9)	–	–	–
Sometimes	60 (13.0)	–	–	–
Often	86 (18.6)	–	–	–
Always	103 (22.3)	–	–	–
Prefer not to answer	8 (1.7)	–	–	–
Modified Monash Model Category for current practice location (*n* (%))				
MM 1—Metropolitan	320 (69.3)	32 (62.8)	–	–
MM 2—Regional	41 (8.9)	6 (11.7)	–	–
MM 3—Large rural town	29 (6.3)	4 (7.8)	–	–
MM 4—Medium rural town	8 (1.7)	0 (0.0)	–	–
MM 5—Small rural town	44 (9.6)	6 (11.8)	–	–
MM 6—Remote community	7 (1.5)	2 (3.9)	–	–
MM 7—Very remote community	9 (2.0)	1 (2.0)	–	–
Prefer not to answer	4 (0.9)	0 (0.0)	–	–

*N* = total responses for that question and population, n = total responses for that answer, % =* n*/*N* × 100%

*A multiple answer question, percentages will not add up to 100

^#^An exclusive answer

– Question was not asked of that respondent group

### Use of the professional practice resources

**Fig. 1:**
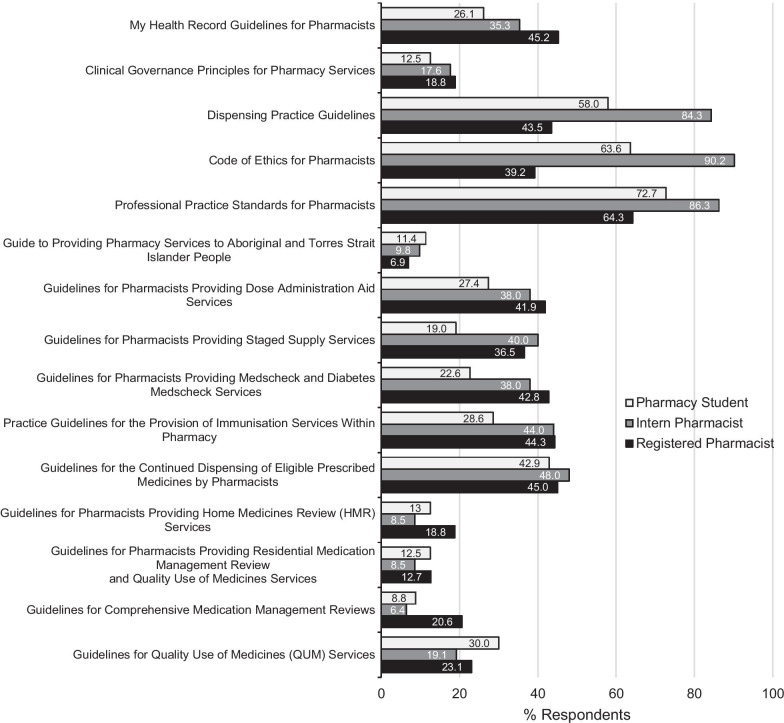
Respondents use of professional practice guidance resources in the past 12 months by respondent group

The proportion of participants who used the selected professional practice guidance resources in the last 12 months by participant group is shown in Fig. 1. Further detail is provided in Table 2. The most frequently used professional practice guidance resources in the past 12 months were the Australian Professional Practice Standards for Pharmacists, followed by the Dispensing Practice Guidelines and the Code of Ethics for Pharmacists (Table 2). A greater proportion of intern pharmacist and pharmacy students used these top three resources compared to registered pharmacists (Fig. 1). All guidance resources except the Australian Professional Practice Standards for Pharmacists had been used by less than 50% of all respondents (Fig. 1, Table 2). A smaller proportion of pharmacy student respondents had used the Core Professional Services Resources (Group 2) compared to registered pharmacists and interns (Fig. 1, Table 2).

**Table 2:** Respondents use of professional practice guidance resources in the last 12 months by respondent group

Professional practice guidance resource	Registered Pharmacist		Pharmacy intern		Pharmacy student		All Respondents	
Resource Group 1—Overarching Practice Standards, Codes and Guidelines	*N* = *462*		*N* = *51*		*N* = *88*		*N* = *601*	
	*n*	%	*n*	%	*n*	%	*n*	%
My Health Record Guidelines for Pharmacists	209,	45.2	18,	35.3	23,	26.1	250,	41.6
Clinical Governance Principles for Pharmacy Services	87,	18.8	9,	17.6	11,	12.5	107,	17.8
Dispensing Practice Guidelines	201,	43.5	43,	84.3	51,	58.0	295,	49.1
Code of Ethics for Pharmacists	181,	39.2	46,	90.2	56,	63.6	283,	47.1
Professional Practice Standards for Pharmacists	297,	64.3	44,	86.3	64,	72.7	405,	67.4
Guide to Providing Pharmacy Services to Aboriginal and Torres Strait Islander People	32,	6.9	5,	9.8	10,	11.4	47,	7.8

Resource Group 2—Community Pharmacy Core Professional Services Practice Guidelines	*N* = *317*		*N* = *36*		*N* = *49*		*N* = *402*	
	*n*	%	*n*	%	*n*	%	*n*	%
Guidelines for Pharmacists Providing Dose Administration Aid Services	188,	41.9	19,	38	23,	27.4	230,	39.5
Guidelines for Pharmacists Providing Staged Supply Services	164,	36.5	20,	40	16,	19.0	200,	34.3
Guidelines for Pharmacists Providing Medscheck and Diabetes Medscheck Services	192,	42.8	19,	38	19,	22.6	230,	39.5
Practice Guidelines for the Provision of Immunisation Services Within Pharmacy	199,	44.3	22,	44	24,	28.6	245,	42.0
Guidelines for the Continued Dispensing of Eligible Prescribed Medicines by Pharmacists	202,	45.0	24,	48	36,	42.9	262,	44.9

Resource Group 3—Accredited Medication Review Services Practice Guidelines	*N* = *317*		*N* = *36*		*N* = *49*		*N* = *402*	
	*n*	%	*n*	%	*n*	%	*n*	%
Guidelines for Quality Use of Medicines (QUM) Services	102,	23.1	9,	19.1	24,	30.0	135,	23.7
Guidelines for Pharmacists Providing Home Medicines Review (HMR) Services	83,	18.8	4,	8.5	10,	13	97,	17.0
Guidelines for Pharmacists Providing Residential Medication Management Review and QUM Services	56,	12.7	4,	8.5	10,	12.5	70,	12.3
Guidelines for Comprehensive Medication Management Reviews	91,	20.6	3,	6.4	7,	8.8	101,	17.8

*N* = total responses for that question and population

*n* = total responses for that answer, e.g., yes I have used this resource in the past 12 months

% = *n*/*N* × 100%

*QUM* Quality Use of Medicines

All Medication Review Resources (Group 3) had been used by less than a quarter of participants in each respondent group. A greater proportion of pharmacy students and registered pharmacists had used each of these resources than intern pharmacists (Fig. 1, Table 2).

Table 3 shows the proportion of respondents’ selected reasons for using the guidance resources, how respondents used the resources, perceived usefulness and reasons for not using professional practice resources by respondent group and resource group.

**Table 3:** Respondents reasons for using the practice guidance resources, how they used the resources, perceived usefulness and reasons for not using professional practice resources by respondent and resource group

Reasons for using professional practice resource(s)*	Resource Group 1—Overarching Practice Standards, Codes and Guidelines								Resource Group 2—Community Pharmacy Core Professional Services Practice Guidelines								Resource Group 3—Accredited Medication Review Services Practice Guidelines							
	Registered Pharmacist		Pharmacy intern		Pharmacy student		All Respondents		Registered Pharmacist		Pharmacy intern		Pharmacy student		All Respondents		Registered Pharmacist		Pharmacy intern		Pharmacy student		All Respondents	
	*N*** = *363***		*N*** = *48***		*N*** = *72***		*N*** = *483***		*N*** = *317***		*N*** = *36***		*N*** = *49***		*N*** = *402***		*N*** = *165***		*N*** = *12***		*N*** = *34***		*N*** = *211***	
	*n*	%	*n*	%	*n*	%	*n*	%	*n*	%	*n*	%	*n*	%	*n*	%	*n*	%	*n*	%	*n*	%	*n*	%
To familiarise myself with the contents	206	56.7	38	79.2	49	68.1	293	60.7	173	54.6	30	83.3	33	67.3	236	58.7	99	60.0	9	75.0	20	58.8	128	60.7
To update my knowledge	196	54.0	26	54.2	42	58.3	264	54.7	202	63.7	24	66.7	27	55.1	253	62.9	110	66.7	7	58.3	14	41.2	131	62.1
To check that my practice is reflective of best practice	188	51.8	27	56.3	36	50.0	251	52.0	192	60.6	18	50.0	14	28.6	224	55.7	90	54.5	5	41.7	7	20.6	102	48.3
For continuing professional development	172	47.4	26	54.2	26	36.1	224	46.4	120	37.9	12	33.3	10	20.4	142	35.3	51	30.9	4	33.3	11	32.4	66	31.3
As a teaching resource for pharmacy students, intern pharmacists, pharmacy/dispensary assistants or colleagues	118	32.5	18	37.5	25	34.7	161	33.3	92	29.0	10	27.8	14	28.6	116	28.9	30	18.2	5	41.7	12	35.3	47	22.3
To resolve a situation while providing a service/patient care	90	24.8	7	14.6	8	11.1	105	21.7	87	27.4	6	16.7	5	10.2	98	24.4	30	18.2	0	0.0	1	2.9	31	14.7
Other^	18	5.0	3	6.3	4	5.6	25	5.2	16	5.0	1	2.8	3	6.1	20	5.0	9	5.5	0	0.0	1	2.9	10	4.7
I cannot remember#	0	0.0	0	0.0	3	4.2	3	0.6	1	0.3	0	0.0	1	2.0	2	0.5	1	0.6	0	0.0	1	2.9	2	0.9

How did they use the selected professional practice resource(s)*	*N* = *363*		*N* = *48*		*N* = *72*		*N* = *483*		*N* = *317*		*N* = *36*		*N* = *49*		*N* = *402*		*N* = *165*		*N* = *12*		*N* = *34*		*N* = *211*	
	*n*	%	*n*	%	*n*	%	*n*	%	*n*	%	*n*	%	*n*	%	*n*	%	*n*	%	*n*	%	*n*	%	*n*	%
Read part of it	236	65.0	38	79.2	59	81.9	333	68.9	174	54.9	26	72.2	33	67.3	233	58.0	80	48.5	9	75.0	23	67.6	112	53.1
Read all of it	93	25.6	15	31.3	10	13.9	118	24.4	132	41.6	11	30.6	12	24.5	155	38.6	71	43.0	4	33.3	6	17.6	81	38.4
Applied the information that I read to adapt my practice	196	54.0	26	54.2	36	50.0	258	53.4	159	50.2	18	50.0	21	42.9	198	49.3	71	43.0	2	16.7	5	14.7	78	37.0
Applied the information that I read to advise others on their practice	69	19.0	5	10.4	1	1.4	75	15.5	108	34.1	9	25.0	7	14.3	124	30.8	23	13.9	0	0.0	1	2.9	24	11.4
Confirmed appropriateness of my current practice	173	47.7	23	47.9	27	37.5	223	46.2	132	41.6	9	25.0	12	24.5	153	38.1	57	34.5	4	33.3	4	11.8	65	30.8
Other^	2	0.6	1	2.1	0	0.0	3	0.6	8	2.5	0	0.0	1	2.0	9	2.2	2	1.2	0	0.0	0	0.0	2	0.9
I can't remember#	3	0.8	1	2.1	1	1.4	5	1.0	0	0.0	0	0.0	1	2.0	1	0.2	1	0.6	0	0.0	1	2.9	2	0.9

Usefulness for group professional practice resource(s) that were used^+^	*N* = *363*		*N* = *48*		*N* = *72*		*N* = *483*		*N* = *317*		*N* = *36*		*N* = *49*		*N* = *402*		*N* = *165*		*N* = *12*		*N* = *34*		*N* = *211*	
	*n*	%	*n*	%	*n*	%	*n*	%	*n*	%	*n*	%	*n*	%	*n*	%	*n*	%	*n*	%	*n*	%	*n*	%
Very—the information contained was exactly what I needed/expected	175	48.2	21	43.8	33	45.8	229	47.4	210	66.2	21	58.3	31	63.3	262	65.2	110	66.7	9	75.0	22	64.7	141	66.8
Some what—some of the information was what I needed/expected	165	45.5	27	56.3	34	47.2	226	46.8	101	31.9	14	38.9	15	30.6	130	32.3	48	29.1	3	25.0	10	29.4	61	28.9
Not at all—the information in this document was not what I needed/expected	5	1.4	0	0.0	0	0.0	5	1.0	3	0.9	1	2.8	0	0.0	4	1.0	3	1.8	0	0.0	0	0.0	3	1.4
I can't remember	18	5.0	0	0.0	5	6.9	23	4.8	3	0.9	0	0.0	3	6.1	6	1.5	4	2.4	0	0.0	2	5.9	6	2.8

Reasons for not using selected professional practice resource(s)^*^	*N* = *462*		*N* = *51*		*N* = *88*		*N* = *601*		*N* = *378*		*N* = *43*		*N* = *75*		*N* = *496*		*N* = *412*		*N* = *46*		*N* = *77*		*N* = *535*	
	*n*	%	*n*	%	*n*	%	*n*	%	*n*	%	*n*	%	*n*	%	*n*	%	*n*	%	*n*	%	*n*	%	*n*	%
I did not know that they existed	120	26.0	35	68.6	32	36.4	187	31.1	15	4.0	17	39.5	23	30.7	55	11.1	51	12.4	21	45.7	24	31.2	96	17.9
I did not know how to access them	81	17.5	18	35.3	32	36.4	131	21.8	19	5.0	14	32.6	14	18.7	47	9.5	22	5.3	8	17.4	14	18.2	44	8.2
They are not relevant to my practice area	148	32.0	6	11.8	17	19.3	171	28.5	188	49.7	12	27.9	17	22.7	217	43.8	235	57.0	24	52.2	27	35.1	286	53.5
I did not need the information that they contain	235	50.9	23	45.1	43	48.9	301	50.1	163	43.1	18	41.9	41	54.7	222	44.8	161	39.1	20	43.5	38	49.4	219	40.9
I do not think the content will be useful, because I already know this information	63	13.6	2	3.9	4	4.5	69	11.5	45	11.9	3	7.0	1	1.3	49	9.9	14	3.4	0	0.0	1	1.3	15	2.8
I do not think it will have the information that I need	51	11.0	6	11.8	6	6.8	63	10.5	16	4.2	2	4.7	3	4.0	21	4.2	6	1.5	0	0.0	1	1.3	7	1.3
I have used these guidelines in the past but not in the last 12 months#	134	29.0	3	5.9	9	10.2	146	24.3	82	21.7	3	7.0	7	9.3	92	18.5	61	14.8	3	6.5	2	2.6	66	12.3
There are more relevant and/or complete and/or useful resources for this information that I use instead^	15	3.2	0	0.0	0	0.0	15	2.5	9	2.4	0	0.0	0	0.0	9	1.8	6	1.5	0	0.0	0	0.0	6	1.1
There are resources for this information that are easier to access^	4	0.9	0	0.0	1	1.1	5	0.8	1	0.3	0	0.0	0	0.0	1	0.2	2	0.5	0	0.0	0	0.0	2	0.4
Other^	13	2.8	1	2.0	4	4.5	18	3.0	6	1.6	2	4.7	2	2.7	10	2.0	3	0.7	1	2.2	2	2.6	6	1.1

*N* = total responses for that question and population

*n* = total responses for that answer

% = *n*/*N* × 100%

^*^Multiple answer question, percentages will not add up to 100

^+^A single answer question, percentages will add up to 100

^#^An exclusive answer

^^^Indicates text response was allowed

### Reasons for using selected professional practice resources

The top three most common reasons for using selected professional practice guidance resources were for respondents to familiarise themselves with the contents, update their knowledge and to check their practice reflects best practice (Table 3).

### How respondents used selected professional practice resources

Most respondents only read part of the selected guidance resource. This was mostly consistent across respondent groups (Table 3).

### Perceived usefulness of selected professional practice resources

Of those who had used at least one professional practice guidance resource most respondents found the resources ‘very useful’ (Table 3). Few respondents indicated the resources were ‘not at all’ useful (Table 3).

### Reasons for *not* using professional practice resources

The most common reasons for not using the professional practice guidance resources were that the respondents did not need the information that the resource contained, the respondents did not know the resource existed or respondents did not considering the resources relevant to their practice area (Table 3). Less than a quarter of respondents indicated that they had used these resources in the past but not in the last 12 months (Table 3).

## Discussion

The current study is the first to investigate if and how pharmacy students, intern pharmacists and registered pharmacists use professional practice guidance resources. All professional practice resources investigated, except the Professional Practice Standards, had been used by less than half of all respondents in the preceding 12 months. The results show differences in use between pharmacy student, intern pharmacist and registered pharmacists’ and suggest some resources may be more relevant to those learning (students and interns) compared to those practising (interns and registered pharmacists). These usage patterns may help to interpret inconsistencies in practice and can inform the tailoring of professional practice resources for future use.

To the researcher’s knowledge this is the first-time changing use of practice guidance resources across pharmacist career stages has been described in the literature. The findings of this study suggest pharmacy students and interns used Overarching Resources that relate to essential components of pharmacy practice more often than pharmacists. These results are not surprising as students and interns need to understand the principles of being a pharmacist in preparation for provisional registration at the end of their degree. Interestingly students were also more likely to say that these resources were only somewhat useful compared to interns and pharmacists. The reasons identified for using professional practice resources was generally consistent across resource and respondent groups. Identified reasons for use included improving familiarity with the resource, updating knowledge, and checking practice. In contrast, variability for reasons for not using the resources were identified between respondent groups. Students and interns more often indicated that they did not know the resource existed compared to pharmacists. All groups indicated to varying degrees they did not need the resources that they had not used or that the practice resource was not relevant to their practice. This observed variability suggests that non-use may be resource dependent or driven by the current role, scope of practice and experience of the respondent.

Limited existing literature on the use of professional practice guidance resources in health professions, and in particular pharmacy, means that limited comparisons can be made with the current study’s findings. Chaar et al. [7] explored the application of ethical principles and use of the Code of Ethics for Australian pharmacists. Hattingh et al. [16] investigated Australian pharmacists’ use of the Practice Standards for Dispensing. Both reported that these resources do not appear to be used by most pharmacists in daily practice. The results of the current the current study are consistent with this. In particular, in the current study only 43.5% of pharmacists reported using the dispensing practice guideline in the previous 12 months, aligning with Hattingh et al.’s finding that the majority of pharmacists did not know about or use the dispensing practice standard to develop their procedures [16]. This lack of awareness and our observed lack of use may explain, at least in part, why the majority of professional transgressions observed in disciplinary hearings related to dispensing processes [6]. Work by Nash et al. [34] on the use of National Competency Standards Framework for Pharmacists by Australian pharmacy students and pharmacists, found there is no accepted measure of what amount of practice resource use is associated with or necessary for quality practice. Given it is a condition of registration each year that pharmacists review the competency standards to identify areas, where they may need targeted professional development, it is alarming that Nash et al. [34] found not all pharmacists in their study had done this. No such requirement exists for the frequency of review for the professional practice resources examined in this study, and opinions on acceptable frequency of use are likely to differ across the profession. However, it would seem reasonable that these practice guidance resources are reviewed at least annually when pharmacists are renewing their registration and planning professional development to ensure they are aware of the content and any updates or changes.

### Implications for practice

Where quality professional service provision and resultant renumeration are dependent on following guidelines, it would seem prudent that the pharmacist is always fully aware of the content of professional practice guidance resources. Continual review by practising pharmacists may be necessary to ensure that they are aware of how to provide acceptable patient care and avoid professional transgressions. Furthermore, if these resources are used during practice and provision of patient care they need to be accessed and read quickly, which has implications for the layout, host platforms and navigation functionalities. If registered pharmacists are only prompted to access guidelines when needing information for providing a service or care that they are unfamiliar with, this may limit their ability to keep up to date if guidance changes in between these prompts. For example, vaccination guidelines are continually changing throughout the national roll out in response to the COVID-19 pandemic and if pharmacists had only reviewed the guidelines at the start of the roll out, they may provide a service that does not meet guidelines. While the use of these resources has not been directly linked to pharmacist performance in providing these services, complying with them is correlated with maintaining registration in the case of the Code of Ethics and Professional Practice Standards [1, 2]. Compliance is also mandated for renumeration for some services (e.g., dose administration aid supply and home medicines review) [17, 26] and adherence to all resources is required to meet legal, ethical and professional practice expectations. Practicing in accordance with all resources closely would ensure a degree of consistency in service provision which serves to maintain trust in the profession from the people it serves [35, 36].

### Recommendations for practice

As usage patterns for professional practice guidance resources differ throughout training to registration, resource developers should consider if these resources need to be tailored to the audience most likely to use them. This could be achieved through in-depth consultation with key stakeholders on what influences their behaviour. A richer understanding of influences for individual groups and resources could facilitate co-design and review of the resources. Professional practice resources need to be adequately detailed for pharmacist interns and for pharmacy students. They need to be easy to locate and navigate for practising pharmacists seeking information to provide immediate patient care. This balance may be hard to strike in a single document; however, with current technology, platforms for hosting professional practice resources could be adaptable and use display logic, diagrams, search functions and hyperlinks to account for this. Issues with awareness of the existence of the resources were also raised and suggest professional practice resource developers should revise how resources are shared and disseminated to the profession and students. The results of this study can directly help to guide the review process for the resources studied and may also be useful for guiding review of pharmacist professional practice resources internationally. Furthermore, these findings may also be useful to other health professions when considering review of their own professional resources.

### Strengths/limitations

This study is the first to explore if and how pharmacists use a large range of professional practice guidance resources. This is one of the largest surveys to be completed involving Australian pharmacy students, interns and pharmacists and offers valuable insights into their usage patterns of professional practice resources. However, given this was a cross sectional survey, a number of limitations may have affected the generalisability of the results. First, common to all survey methodology completion of the study may be subject to respondent bias, where only motivated eligible participants respond. Thus, the results may have been influenced by limited responses from dis-engaged practitioners. We attempted to address that through a multi-faceted recruitment and distribution strategy; however, participation remained voluntary. It is possible that the results were skewed by voluntary participation, though it is likely that the findings reported here are more conservative findings compared to if disengaged practitioners had responded. The researchers extensively advertised the survey on a range of different platforms. This led to over 1% of each population completing the survey and a largely representative pharmacist sample when compared to national registration statistics. The participants seem to broadly represent those of the profession when compared to previous studies and current Australian Pharmacy Board data specifically for demographic characteristics, such as age and gender, and practice location [33, 37]. Differences of note are that the proportion of registered pharmacists’ participants in Western Australia were higher and New South Wales were lower than those of the reported population. Demographic characteristics broadly reflect the profession though do not reflect professional engagement and motivation. The results may also be subject to retrospective recall bias. However, participants did have the option to select ‘I cannot remember’ if this was the case and its reasonable to assume that if they do not remember using the resource they probably did not. Furthermore, the self-reported use of a guideline was not compared to an objective assessment on whether it was required so no conclusions can be reached as to appropriateness of the frequency of reported use. This study was conducted during the height of Australia’s second COVID-19 pandemic wave, when pharmacists were under considerable stress and regulations and guidelines for services were changing rapidly. How this may have affected the results is unknown. The present study shows a snapshot of use over 12 months by the respondents limiting understanding of what ‘lifetime’ use may be.

### Recommendations for future research

The notion behind respondents expressing they did not ‘need’ particular guidance resources would be interesting to explore further. If it is that respondents assume they know the information, how do they know this knowledge is up to date? Does this assumption align with the resource developers’ expectations? Does the pharmacist’s knowledge translate to their practice? If resources users perceive the information to be irrelevant, how could it be improved for future use? To address this future work should set out to elicit a deeper understanding of why these resource use patterns were observed and what influences this professional behaviour. Qualitative methods may be particularly helpful here. This information could serve to inform redesign of the resources or behavioural interventions to enhance their usability.

## Conclusions

Australian pharmacy students, intern pharmacists and registered pharmacists use of professional practice guidance resources varies. Core overarching resources are mostly used by pharmacy students and intern pharmacists compared to practising pharmacists, whereas interns and practising pharmacists are more likely, than pharmacy students to use guidelines that pertain to services that they are involved in providing (e.g., dose administration aids). Common reasons for using professional practice guidance resources included for familiarisation with the content, to seek information, and to check and support best practice. However, limited awareness of the existence of some resources and a lack of perceived relevance to individual practice were the most identified reasons for not using professional practice resources. This may pose a risk for individuals failing to meet professional obligations and result in professional transgressions that negatively impact patient experience or care. These results suggest that professional practice guidance resources need to be adequately detailed for new pharmacists and those studying, and easily accessed and navigated for practising pharmacists seeking information to provide immediate patient care. The results of this study are, therefore, invaluable to professional bodies responsible for developing these resources, who should consult key stakeholder groups (e.g., pharmacy students, intern pharmacists and registered pharmacists) in the resource design process. A richer understanding of what influences the use of professional practice resources could support the development of tailored interventions to increase professional behaviour across different groups and contexts and assist individuals to meet professional obligations.

## Data Availability

The data sets used and/or analysed during the current study may be available from the corresponding author conditionally in line with appropriate ethical approval.

## Appendix

See Table 4.

**Table 4:** Checklist for Reporting Results of Internet E-Surveys (CHERRIES)

Checklist Item	Explanation	Manuscript section
Describe survey design	Describe target population, sample frame. Is the sample a convenience sample? (In “open” surveys this is most likely.)	See “Methods” section, subheadings; ‘Study Design’, ‘Participants’ and ‘Sample Size.’
IRB approval	Mention whether the study has been approved by an IRB	See “Methods” section, subheading ‘Ethical Approval.’
Informed consent	Describe the informed consent process. Where were the participants told the length of time of the survey, which data were stored and where and for how long, who the investigator was, and the purpose of the study?	See “Methods” section, sub-heading ‘Final Survey’ paragraph 3
Data protection	If any personal information was collected or stored, describe what mechanisms were used to protect unauthorized access	See “Methods” section, sub-heading ‘Final Survey’ paragraph 2. All responses were anonymous
Development and testing	State how the survey was developed, including whether the usability and technical functionality of the electronic questionnaire had been tested before fielding the questionnaire	See “Methods” section, sub-heading ‘Survey Design for question development and piloting process
Open survey versus closed survey	An “open survey” is a survey open for each visitor of a site, while a closed survey is only open to a sample which the investigator knows (password-protected survey)	The survey was not password protected and screening questions at the beginning of the survey should prevent ineligible responses
Contact mode	Indicate whether or not the initial contact with the potential participants was made on the Internet. (Investigators may also send out questionnaires by mail and allow for Web-based data entry.)	See “Methods” section, sub-heading ‘Recruitment/Distribution.’
Advertising the survey	How/where was the survey announced or advertised? Some examples are offline media (newspapers), or online (mailing lists—If yes, which ones?) or banner ads (where were these banner ads posted and what did they look like?). It is important to know the wording of the announcement as it will heavily influence who chooses to participate. Ideally the survey announcement should be published as an appendix	See “Methods” section, sub-heading ‘recruitment/distribution.’
Web/E-mail	State the type of e-survey (e.g., one posted on a Web site, or one sent out through e-mail). If it is an e-mail survey, were the responses entered manually into a database, or was there an automatic method for capturing responses?	See “Methods” section, sub-heading ‘Recruitment/Distribution’ the survey was hosted through an online platform that automatically recorded responses and was accessible through a weblink
Context	Describe the Web site (for mailing list/newsgroup) in which the survey was posted. What is the Web site about, who is visiting it, what are visitors normally looking for? Discuss to what degree the content of the Web site could pre-select the sample or influence the results. For example, a survey about vaccination on a anti-immunization Web site will have different results from a Web survey conducted on a government Web site	NA—not hosted on a website
Mandatory/voluntary	Was it a mandatory survey to be filled in by every visitor who wanted to enter the Web site, or was it a voluntary survey?	NA—not hosted on a website
Incentives	Were any incentives offered (e.g., monetary, prizes, or non-monetary incentives such as an offer to provide the survey results)?	See “Methods” section, sub-heading ‘Incentive To Participate’
Time/Date	In what timeframe were the data collected?	See “Methods” section, sub-heading ‘Recruitment/Distribution.’
Randomization of items or questionnaires	To prevent biases items can be randomized or alternated	See “Methods” section, sub-heading ‘Final Survey’ paragraph 1. Response items were randomized
Adaptive questioning	Use adaptive questioning (certain items, or only conditionally displayed based on responses to other items) to reduce number and complexity of the questions	See “Methods” section, sub-heading ‘Final Survey’ paragraph 1
Number of Items	What was the number of questionnaire items per page? The number of items is an important factor for the completion rate	See “Methods” section, sub-heading ‘Final Survey’ paragraph 1. This varied due to adaptive questioning being enabled
Number of screens (pages)	Over how many pages was the questionnaire distributed? The number of items is an important factor for the completion rate	See “Methods” section, sub-heading ‘Final Survey’ paragraph 1
Completeness check	It is technically possible to do consistency or completeness checks before the questionnaire is submitted. Was this done, and if “yes”, how (usually JAVAScript)? An alternative is to check for completeness after the questionnaire has been submitted (and highlight mandatory items). If this has been done, it should be reported. All items should provide a non-response option such as “not applicable” or “rather not say”, and selection of one response option should be enforced	See “Methods” section, sub-heading ‘Final Survey’ paragraph 2
Review step	State whether respondents were able to review and change their answers (e.g., through a Back button or a Review step which displays a summary of the responses and asks the respondents if they are correct)	See “Methods” section, sub-heading ‘Final Survey’ paragraph 2
Unique site visitor	If you provide view rates or participation rates, you need to define how you determined a unique visitor. There are different techniques available, based on IP addresses or cookies or both	Not reported—data not collected
View rate (Ratio of unique survey visitors/unique site visitors)	Requires counting unique visitors to the first page of the survey, divided by the number of unique site visitors (not page views!). It is not unusual to have view rates of less than 0.1% if the survey is voluntary	Not reported—data not collected
Participation rate (Ratio of unique visitors who agreed to participate/unique first survey page visitors)	Count the unique number of people who filled in the first survey page (or agreed to participate, for example by checking a checkbox), divided by visitors who visit the first page of the survey (or the informed consents page, if present). This can also be called “recruitment” rate	Not reported—data not collected
Completion rate (Ratio of users who finished the survey/users who agreed to participate)	The number of people submitting the last questionnaire page, divided by the number of people who agreed to participate (or submitted the first survey page). This is only relevant if there is a separate “informed consent” page or if the survey goes over several pages. This is a measure for attrition. Note that “completion” can involve leaving questionnaire items blank. This is not a measure for how completely questionnaires were filled in. (If you need a measure for this, use the word “completeness rate”.)	Completion rate for the full survey (includes data not reported in this study) was calculated as the number of respondents who completed/respondents who consented (554/764, 72.5%)
Cookies used	Indicate whether cookies were used to assign a unique user identifier to each client computer. If so, mention the page on which the cookie was set and read, and how long the cookie was valid. Were duplicate entries avoided by preventing users access to the survey twice; or were duplicate database entries having the same user ID eliminated before analysis? In the latter case, which entries were kept for analysis (e.g., the first entry or the most recent)?	See “Methods” section, sub-heading ‘Final Survey’ paragraph 4
IP check	Indicate whether the IP address of the client computer was used to identify potential duplicate entries from the same user. If so, mention the period of time for which no two entries from the same IP address were allowed (e.g., 24 h). Were duplicate entries avoided by preventing users with the same IP address access to the survey twice; or were duplicate database entries having the same IP address within a given period of time eliminated before analysis? If the latter, which entries were kept for analysis (e.g., the first entry or the most recent)?	See “Methods” section, sub-heading ‘Final Survey’ paragraph 4
Log file analysis	Indicate whether other techniques to analyze the log file for identification of multiple entries were used. If so, please describe	NA
Registration	In “closed” (non-open) surveys, users need to login first and it is easier to prevent duplicate entries from the same user. Describe how this was done. For example, was the survey never displayed a second time once the user had filled it in, or was the username stored together with the survey results and later eliminated? If the latter, which entries were kept for analysis (e.g., the first entry or the most recent)?	NA
Handling of incomplete questionnaires	Were only completed questionnaires analyzed? Were questionnaires which terminated early (where, for example, users did not go through all questionnaire pages) also analyzed?	See “Methods” section, sub-heading ‘Data Analysis.’
Questionnaires submitted with an atypical timestamp	Some investigators may measure the time people needed to fill in a questionnaire and exclude questionnaires that were submitted too soon. Specify the timeframe that was used as a cutoff point, and describe how this point was determined	See “Methods” section, sub-heading ‘Data Analysis.’ The full survey required 15–20 min to complete
Statistical correction	Indicate whether any methods such as weighting of items or propensity scores have been used to adjust for the non-representative sample; if so, please describe the methods	NA

This checklist has been modified from Eysenbach G. Improving the quality of Web surveys: the Checklist for Reporting Results of Internet E-Surveys (CHERRIES). J Med Internet Res. 2004 Sep 29;6(3):e34 [erratum in J Med Internet Res. 2012; 14(1): e8.]. Article available at https://www.jmir.org/2004/3/e34/; erratum available https://www.jmir.org/2012/1/e8/. Copyright ©Gunther Eysenbach. Originally published in the Journal of Medical Internet Research, 29.9.2004 and 04.01.2012

## Abbreviation

PSA: Pharmaceutical Society of Australia
